# Trends in Incidence of Subtrochanteric Fragility Fractures and Bisphosphonate Use Among the US Elderly, 1996–2007

**DOI:** 10.1002/jbmr.233

**Published:** 2010-09-02

**Authors:** Zhong Wang, Timothy Bhattacharyya

**Affiliations:** Intramural Research Program, National Institute of Arthritis, Musculoskeletal and Skin Diseases, National Institutes of HealthBethesda, MD, USA

**Keywords:** Aging, Bone, Population Studies, Osteoporosis, Bisphosphonates

## Abstract

Increasing numbers of atypical hip fractures have been reported among patients with bisphosphonate use. However, the nature and extent of the problem are unknown despite recent investigations. To analyze national trends in hip fractures and medication use in the elderly US population, we respectively used the Nationwide Inpatient Sample (NIS) and the Medical Expenditure Panel Survey (MEPS) from 1996 to 2007. In NIS, subtrochanteric fragility fractures were compared with typical hip fractures in femoral neck and intertrochanteric regions. Between 1996 and 2007, age-adjusted rates for typical hip fractures decreased by 31.6% among women (from 1020.5 to 697.4 per 100,000 population) and 20.5% among men (from 424.9 to 337.6 per 100,000 population). In contrast, overall trends in age-adjusted rates for subtrochanteric fragility fractures remained unchanged among men (*p* = .34) but increased 20.4% among women from 28.4 (95% confidence interval [CI] 27.7–29.1) in 1999 to 34.2 (95% CI 33.4–34.9) per 100,000 population in 2007. The annual percentage increase was 2.1% (95% CI 1.3–2.8, *p* < .001) based on joinpoint regression analysis. In MEPS, bisphosphonate use increased predominantly in women (from 3.5% in 1996 to 16.6% in 2007) compared with men (2.3% in 2007). In the context of declining typical hip fractures among the US elderly, we observed small but significant increases in the incidence of subtrochanteric fragility fractures from 1999 among postmenopausal women. Using age-adjusted rates, we estimated that for every 100 or so reduction in typical femoral neck or intertrochanteric fractures, there was an increase of one subtrochanteric fragility fracture. © 2011 American Society for Bone and Mineral Research.

## Introduction

In the United States, there are approximately 320,000 people hospitalized for hip fractures each year,(1) with a 20% mortality rate in the following year.(2) Hip fractures occur mostly in women over 60 years of age who were at risk of developing osteoporosis after menopause. Osteoporosis is characterized as an increased skeletal fragility and microarchitectural deterioration in the bones.(3) Prevention and treatment of osteoporosis are effective ways to prevent hip fractures. Since 1996 after Food and Drug Administration (FDA) approval, bisphosphonates have been prescribed increasingly to those diagnosed with osteoporosis.(4) Coincident with increased use of bisphosphonates, declines in the rates of hip fractures were observed in Canada(5) and the United States.(6)

Hip fractures typically occur in the femoral neck and intertrochanteric regions but infrequently in subtrochanteric region.(7) Recently, an increasing number of clinical reports suggested an association of atypical hip fractures in subtrochanteric and diaphyseal regions with long-term use of bisphosphonates,(8–17) possibly owing to prolonged suppression of bone turnover.(9,18) However, post-hoc analyses of clinical trial data(19) and a cohort study(20) provided counterevidence. Since these atypical hip fractures are very rare, epidemiologic studies with national-level data seem to be the only data source with enough study power. Indeed, a recent epidemiologic study showed that between 1996 and 2006, the incidence of femur fractures distal to the lesser trochanter remained stable, whereas more proximal hip fractures declined.(21)

In this trend study, we analyzed prevalence in national use of fracture-related medicines in nationally representative surveys while evaluating trends in incidence and total estimates of hip fractures in nationwide inpatient samples during 1996–2007.

## Materials and Methods

### Data sources and samples

The Nationwide Inpatient Sample (NIS) is a nationally representative all-payer inpatient database developed from the Healthcare Cost and Utilization Project (HCUP) by the Agency for Healthcare Research and Quality (AHRQ) and collected annually as a stratified sample from 20% US nonfederal community hospitals. It contains more than 6 million deidentified hospital discharges each year, with the first of up to 15 diagnoses or procedure codes listed as the principal reason for hospitalization or primary procedure incurred, respectively. It also has patient demographic information, with race as self-reported, and hospital characteristic and facility charges related to inpatient stays. We used NIS supplemental trend files, if necessary, which allow accurate comparison from year to year and adjust for changes in sampling frames. We selected data from 1996 to 2007 to evaluate the trends in hospitalizations for hip fractures. Numbers of annual discharges were weighted to generate national estimates for each year. It should be mentioned that medication use was not captured in NIS. Comorbidity category and index were generated with AHRQ software.(22)

The Medical Expenditure Panel Survey (MEPS) is a series of annual large-scale surveys of the civilian noninstitutionalized population across the United States. Its main household component (HC) collects data from a nationally representative subsample of households that participated in the prior year's National Health Interview Survey (conducted by the National Center for Health Statistics). The MEPS contains information about respondents' demographics, insurance status, and health care utilization, including prescription drug use. The MEPS uses both household interviews and pharmacy surveys for its prescribed medicine files. We studied national utilization of medications with bone effects such as bisphosphonates,(19) glucocorticoids,(23) proton-pump inhibitors,(24) statins,(25,26) and beta blockers.(27)

Because the data used were publicly available and contained only deidentified information, the study was exempted by the institutional review boards at NIH, and AHRQ granted the use of NIS and MEPS data.

### Outcome measures

Hip fractures require hospitalizations and surgical interventions, which are captured in NIS data with the *International Classification of Diseases,* Ninth Revision, *Clinical Modification* (ICD-9-CM) codes. We excluded open hip fractures because they are more related to trauma or high-energy impact. After excluding these open hip fractures, closed hip fractures constitute more than 99.5% of all hospitalizations for hip fractures and are likely to be osteoporotic in nature. We defined typical hip fractures as hospitalizations with a primary diagnosis of closed femoral neck fractures (ICD-9-CM 820.00, 820.01, 820.02, 820.03, 820.09, and 820.8) or closed intertrochanteric fractures (ICD-9-CM 820.21) and subtrochanteric fragility fractures as hospitalizations with a primary diagnosis of closed subtrochanteric fractures (ICD-9-CM 820.22). Furthermore, we limited the records to those with a concurrent primary ICD-9-CM procedure code for primary surgical treatment (7855, 7905, 7915, 7925, 7935, 7945, 7955, 7965, 7975, 7985, and 7995) or replacement (0074, 0075, 0076, 0077, 0085, 0086, 0087, 8151, 8152, and 8169). In this way, we captured over 90% of original records and estimated incidences of hip fractures and excluded discharges for follow-up visits, procedures for periprosthetic fractures, and revision procedures.

We abstracted data on annual discharge records pertaining to specific types of hip fractures for patients aged 65 years and older, excluding those with missing data on gender (<0.01%). Secondary outcomes included age-standardized proportion of specific hip fractures among total hip fractures from NIS data and percentage of respondents with at least one prescription medication from MEPS.

### Data analysis

To calculate annual hospitalization rates, we used weighted frequencies of hip fractures as the numerator and annual midyear Census population estimates for the older adult population 65 years of age and older as the denominator, and trends in hip fracture rates were stratified by gender and adjusted to the 1996 population in 5-year age groups (65 to 69, 70 to 74, 75 to 79, 80 to 84, and 85+ years old). Standard errors were calculated along with age standardization.(28) For calculation of the proportion of subtrochanteric hip fractures among all hip fractures, the denominator was the total number of hip fractures with ICD-9-CM = 820.X and related primary procedures for hip replacement or treatment. For the analysis of linear trends, we used joinpoint regression analysis to identify significant change over time in the linear regression of the trends in hip fracture rates (Joinpoint Regression Program, Version 3.4.2, October 2009; Statistical Research and Applications Branch, National Cancer Institute, Bethesda, MD, USA). Joinpoint regression analysis enables us to test whether an apparent change in trend or proportion was statistically significant using a Monte Carlo permutation method and estimated variance for each data point.(29)

We used SAS 9.2 (SAS Institute, Inc., Cary, NC, USA) and SUDAAN 10 (Research Triangle Institute, Research Triangle Park, NC, USA). All estimates of fracture proportion and medication use took into consideration the stratified survey design of both NIS and MEPS. To compare national estimates and demographics, a *z* test and chi-square test were used, respectively. Statistical tests were performed at two-sided significance level ≤ .05.

## Results

Table 1 shows the demographic characteristics of women and men over 65 years of age who were hospitalized with subtrochanteric and other hip fractures from 1996 to 2007. Among women, there were 16,766 and 445,991 subtrochanteric and typical hip fractures, respectively. In comparison, there were 5,488 and 141,031 corresponding fractures among men. Patients with subtrochanteric fractures were significantly younger than those with typical hip fractures, with a higher percentage of subtrochanteric fractures in the 65- to 74-year age group. There seemed to be more patients of minority ethnicity with subtrochanteric fractures regardless of gender. Women with subtrochanteric fractures tended to have three or more comorbid conditions. All patients were covered mostly by public insurance, more likely to be white, and were admitted from the emergency room in over 80% of patients. With all the above-mentioned comparisons being significant, there was no detectable difference in the geographic distribution between the two types of fractures (*p* > .05).

**Table 1:** Patient Characteristics

	No. (%) of Women		No. (%) of Men	
		
	Subtrochanteric (n=16,766)	Typical (n=445,991)	Subtrochanteric (n=5,488)	Typical (n=141,031)
Age, y				
65–74	2741 (16)	61549 (14)	1475 (27)	28135 (20)
75–84	6564 (39)	184498 (41)	2366 (43)	62654 (44)
85+	7461 (45)	199944 (45)	1647 (30)	51242 (36)
Race				
White	11263 (67)	307099 (69)	3652 (67)	96859 (68)
Black, Hispanic, Pacific Islander, or Asian	1457 (8)	31360 (7)	522 (9)	11666 (8)
Unknown	4046 (24)	107532 (24)	1314 (24)	33506 (24)
Comorbid diseases				
Congestive heart failure	2872 (17)	71859 (16)	989 (18)	26569 (19)
Chronic pulmonary disease	2779 (17)	79425 (18)	1337 (24)	38127 (27)
Diabetes	3231 (19)	68543 (15)	1261(23)	27058 (19)
Renal Failure	610 (4)	13079 (3)	345 (6)	9927 (7)
Rheumatoid arthritis	324 (2)	8969 (2)	52 (1)	1676 (1)
Depression	1221 (7)	35839 (8)	217 (4)	7992 (6)
No. of comorbidities				
0	1515 (9)	45262 (10)	593 (11)	13333 (9)
1	3749 (22)	105183 (24)	1280 (23)	31285 (22)
2	4481 (27)	124820 (28)	1460 (27)	38321 (27)
> = 3	7021 (42)	170726 (38)	2155 (39)	59092 (42)
Admission Type				
Emergency	14299 (85)	376212 (84)	4674	118253 (83)
Other	981 (6)	32544 (7)	302	10675 (8)
Unknown	1486 (9)	37235 (8)	512	13103 (9)
Primary Payer				
Medicare	15259 (91)	408669 (92)	4774	127915 (90)
Medicaid	181 (1)	4142 (1)	45	1163 (1)
Private	1120 (7)	27609 (6)	520	10328 (7)
Uninsured	77 (0)	2052 (0)	38	692 (0)
Other	129 (1)	3519 (1)	111	1933 (1)
Hospital type				
Teaching	10637 (63)	291722 (65)	3315	92842 (65)
Nonteaching or unknown	6129 (36)	154279 (34)	2173	49189 (35)
Hospital location				
Northeast	3237 (19)	86789 (19)	999	25812 (18)
Midwest	4082 (24)	107757 (24)	1293	34320 (24)
South	6467 (39)	172288 (39)	2096	54078 (38)
West	2980 (18)	79157 (18)	1100	27821 (20)

*Note:* Unweighted numbers are from the Nationwide Inpatient Samples 1996–2007 for patients 65 years of age or older. Characteristics are number (percentage) and percentages may not sum to 100 because of rounding. All comparisons of baseline characteristics (except hospital location) between subtrochanteric and typical hip fractures are significant with a *p* value of less than .001.

### National estimates of hip fractures

**Fig. 1:**
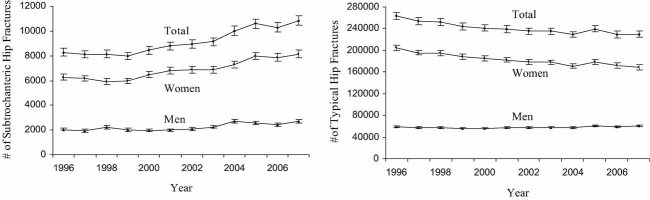
National estimate of subtrochanteric and typical hip fractures in patients aged 65 and older. Data are based on Nationwide Inpatient Samples. Error bars indicate standard deviation.

To understand the disease burden and provide the numerators for the incidence rate, we calculated the annual national estimates of typical and subtrochanteric hip fractures for female, male, and total elderly populations (Fig. 1). For the total elderly population, estimates for hip fractures decreased 11.0% from 275,151 (95% CI 262,262–288,040) in 1996 to 244,889 (95% CI 232,472–257,305) in 2007. Typical hip fractures were 12.8% less in 2007 (229,942, 95% CI 218,224–241,660) than in 1996 (263,623, 95% CI 251,227–276,019) and became significantly lower since 1999. However, the number of subtrochanteric hip fractures increased 31.2% from 8273 (95% CI 7663–8882) in 1996 to 10,853 (95% CI 10,115–11,589) in 2007 and became significantly higher since 2004 (*p* < .05).

### Age-adjusted hip fracture rates

**Fig. 2:**
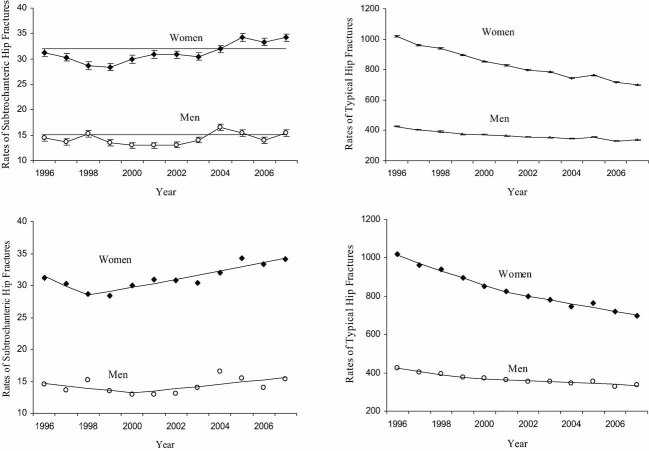
Age-adjusted rates of subtrochanteric and typical hip fractures in elderly patients aged 65 and older. Data are based on Nationwide Inpatient Samples. Error bars indicate 95% confidence interval. Two separate regression lines were drawn through joinpoints based on the best model fit.

The age-adjusted hospitalization rates for hip fractures are shown in Fig. 2. In women, hospitalization rates decreased 31.7% (from 1020.5 to 697.4 per 100,000) for typical hip fractures. On the other hand, for subtrochanteric hip fractures, rates increased 9.6% from 31.2 per 100,000 (95% CI 30.4–32.0) in 1996 to 34.2 per 100,000 (95% CI 33.4–34.9) in 2007. In men, hospitalization rates decreased 18.4% and 20.4% for overall and typical hip fractures, respectively, whereas those for subtrochanteric hip fractures remained unchanged (14.5 per 100,000, 95% CI 13.9–15.1) in 1996 compared with 15.4 per 100,000 (95% CI 14.8-16.0) in 2007.

Considering the segmental nature of linear trends in incidence rates, we used joinpoint regression to analyze changes in rates, with one joinpoint that indicated a change in the linear trend (Fig. 2). The incidence rate for typical hip fractures among women decreased 4.1% per year before and 2.6% per year after the joinpoint at 2001, and among men, 4.1% and 1.4%, respectively (Table 2). For subtrochanteric hip fractures, the rates of decrease from 1995 to 1999 were not significant for either women or men (*p* > .05). However, the annual percentage change since 1999 in age-adjusted rate of subtrochanteric hip fractures was significant for women (2.1%, 95% CI 1.3–2.8) but not for men following at later joinpoint (2.4%, 95% CI –1.1–6.0, *p* = .15).

**Table 2:** Annual Percentage Change (APC) in Age-adjusted Rates of Hip Fractures by Sex According to Joinpoint Regression Analysis

	Overall Hip Fractures		Typical Hip Fractures		Subtrochanteric Hip Fractures	
			
	Segments	APC, %(95% CI)	Segments	APC, %(95% CI)	Segments	APC, %(95% CI)
Both Sexes	1996–2000	−4.1(−5.5 to −2.6)	1996–2000	−4.2(−5.7 to −2.8)	1996–1999	−2.8(−7.1 to 1.7)^c^
	2000–2007	−2.4(−3.0 to −1.7)	2000–2007	−2.6(−3.4 to −2.4)	1999–2007	2.0(1.1 to 2.9)
Female	1996–2001	−3.9(−5.0 to −2.9)	1996–2001	−4.1(−5.1 to −3.1)	1996–1998	−2.9(−12.6 to 3.7)^c^
	2001–2007	−2.4(−3.2 to −1.5)	2001–2007	−2.6(−3.4 to −1.8)	1998–2007	2.1(1.3 to 2.8)
Male	1996–1999	−4.0(−6.9 to −1.0)^a^	1996–1999	−4.1(−6.9 to −1.2)	1996–2000	−2.6(−10.7 to 6.2)^c^
	1999–2007	−1.3(−1.9 to −0.6)^b^	1999–2007	−1.4(−2.1 to −0.7)	2000–2007	2.4(−1.1 to 6.0)^c^

APC = annual percentage change; CI = confidence interval.

a *p* = .017.

b *p* = .003.

c *p* > .05. All other p values are less than .001.

### Age-standardized proportion of subtrochanteric hip fractures

**Fig. 3:**
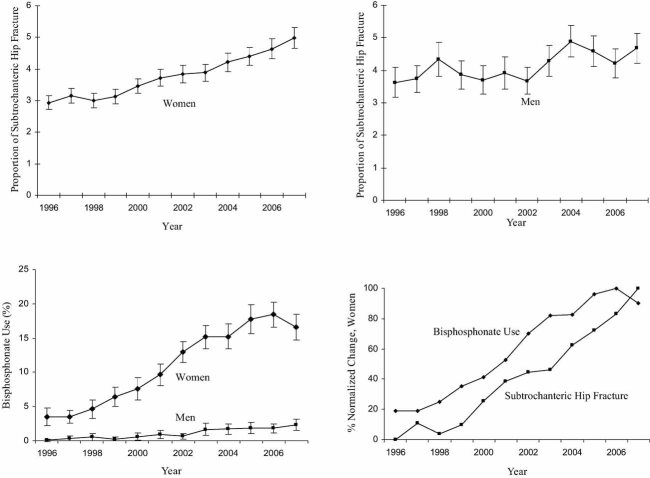
Age-standardized proportion of subtrochanteric hip fractures among all hip fractures and prevalence of bisphosphonate use. Data are based on Nationwide Inpatient Samples for proportions and Medical Expenditure Panel Survey for the medication use. Error bars indicate 95% confidence interval. Percentage changes were normalized in women for the temporal correlation between the bisphosphonate use and subtrochanteric hip fractures.

The trend of changes in proportion of subtrochanteric hip fractures among all hip fractures also was analyzed (Fig. 3). Based on joinpoint regression analysis, the increase in the proportion of subtrochanteric hip fractures among women was significant from 1999 to 2007 [from 2.9% (95% CI 2.7–3.1) in 1996 to 5.0% (95% CI 4.7–5.3) in 2007, *p* < .001]. Among men, no significant changes were seen (*p* = .9 from 1996 to 2001 and *p* = .10 from 2001 to 2007).

### Medication use

From 41,750 respondents aged 65 years or older within MEPS, we obtained the proportion of those who had at least one prescription for bisphosphonates during annual surveys from 1996 to 2007 (Fig. 3). The prevalence of bisphosphonate use significantly increased, particularly among women (from 3.5% in 1996 to 18.4% in 2006, *p* < .001) but less in men (up to 2.3% in 2007). The percent increases were normalized from 0% to 100% for both bisphosphonate use and subtrochanteric hip fractures among women (Fig. 3), indicating that the prevalence of bisphosphonate use preceded the increase in the proportion of subtrochanteric fractures. In contrast, prevalence in national uses of other fracture-related medications such as glucocorticoids, proton-pump inhibitors, statins, and beta blockers increased in both women and men, but the difference is not significant, except the case of statin use, which was more prevalent in men (Supplemental Fig. S1).

## Discussion

In this 12-year study of over 90 million hospital discharge records, we took advantage of the large sample size and nationwide sampling of NIS to estimate rare incidences of subtrochanteric fragility fractures in the elderly population. In the context of a decline in typical hip fractures in both women and men, we found increasing trends in incidence rate and percentage of subtrochanteric hip fractures only among women. The incidence rates of subtrochanteric fractures in women were twice as high as those in men, although the proportions were similar and low (2% to 4%) in the beginning. The secular trend of increases in both rate and proportion were significant in women but not in men, suggesting that elderly women bear increased burdens of such rare fractures not only in absolute terms but also relative to overall hip fractures. Furthermore, these trends since 1999 were preceded by an increase in prevalence of bisphosphonate use among women since 1996. On the other hand, national uses of other fracture-related medications were similar between men and women.

These findings suggest that the case reports of more than 54 atypical hip fractures in the United States since 2005 may be not anecdotal but rather reflective of systemic changes in the nature of hip fractures. This is in agreement with a recent case-control study confirming the association between atypical hip fractures and bisphosphonate use.(30) However, this is contradictory to recent reports from post-hoc analyses of clinical trial data(19) and a cohort study.(20) In both studies, no significant differences were found between typical and subtrochanteric or diaphyseal hip fractures.

Interestingly, in the study by Black and colleagues, point estimates for the risk ratio of subtrochanteric and diaphyseal fractures from three trials were all above 1.00 in the treatment group and higher in trials with longer exposure to bisphosphonates or with more potent agents such as zoledronic acid. It also should be mentioned that in the only trial where the intertrochanteric fractures were significantly reduced (HORIZON trial), the risk ratio of subtrochanteric and diaphyseal femur fractures was highest in point estimates. Furthermore, while seven subtrochanteric or diaphyseal femur fractures in treatment all were found to result from minimal trauma (a fall from standing height or less), all three in the placebo group were caused by higher impact such as falls from a stair, step, or curb. Although it is hard to discuss the significance of these findings, randomized trials should give some indications as to the tendency of risk changes.

In another study with population-based registry, fracture risks for the hip and subtrochanteric or diaphyseal femur were more common in bisphosphonate users than in nonusers.(20) This may be an example of confounding by indication often seen in observational studies,(31) given the tendency to use bisphosphonates in patients with osteoporosis and/or higher risk of hip fractures. In addition, this study did not exclude fractures owing to high-energy trauma. And finally, despite from a national register, fewer than 200 people were determined to be highly adherent with more than 6 years of bisphosphonate treatment.

A recent study of National Hospital Discharge Survey (NHDS) indicates that although there was a decline in hip fracture rate, no increase in closed subtrochanteric fractures was found.(21) This is not unexpected because estimates for less common procedures and diagnoses tend to be less reliable if the sample size is smaller than a million.(32) NIS, on the other hand, has been used for studying other rare medical events such as pneumonia hospitalizations complicated by empyema in children with an incidence rate of less than 10 per 100,000.(33)

Our estimates of rates and declines for typical hip fractures are consistent with earlier studies using NIS(34) and Medicare data.(6) In addition to the diagnosis code, we also incorporated procedure codes to ascertain the incidence of hip fractures and excluded revision surgeries and follow-up visits. Our estimates of subtrochanteric hip fractures were comparable with those from Black and colleagues(19) and Nieves and colleagues,(21,35) although they included femur shaft fractures and had patients from 50 years and older. The reason we did not include the shaft fractures was that we intended to study the proportion of subtrochanteric hip fracture among all hip fractures, and femur shaft fractures represent a different type in that regard. On including the shaft fractures, increasing trends of subtrochanteric and femoral shaft fractures were still observed (unpublished data).

While our results are consistent with the protective effect of bisphosphonates against typical hip fracture, we also showed a temporal correlation between national use of bisphosphonates and delayed increase in subtrochanteric fractures. It remains to be seen whether the increasing incidence should occur specifically among those with relevant medication usage. We noted that women were the predominant users of bisphosphonates and experienced the vast majority of subtrochanteric fractures. If the rise in subtrochanteric fractures were due to an environmental effect, a shift in coding practices, or rising general awareness, it would be seen in men as well.

Our study has strengths related to the national representation, high sensitivity, and contextual analysis for different outcomes. However, there are limitations associated with this ecologic investigation. First, MEPS and NIS may not be representative of the same segment of the US population such that comparing trends in these two data sets has limitations. Second, data were not available or shown regarding other trends in lifestyle factors that also might explain fracture trends, such as physical activity, use of dietary supplements, smoking, and use of other medications such as selective estrogen receptor modulator (SERMs), parathyroid hormone, and estrogen. And finally, the administrative data we used are limited in that closed subtrochanteric fracture were unspecific for those “atypical” fractures without knowing if such characteristic radiographic findings were present as reported in clinical case reports.(14,16,35) We may have overestimated the extent of such fractures by using ICD-9 codes rather than radiographs to identify fractures. However, since subtrochanteric hip fracture is a very rare outcome, claims data may be better suited to address the problem with sufficient power. Our preliminary findings call for a large cohort study with longitudinal data about health outcomes and long-term medication history.

Our data may define the extent of divergence between these two trends. National estimates of hospitalizations for hip fractures declined by more than 30,000 between 1996 and 2007 despite aging of the population. In contrast, there were approximately 2,500 more subtrochanteric hip fractures in 2007 than in 1996. It is obvious that the extent of increase in subtrochanteric hip fractures remains very small compared with the substantial declines in typical or overall hip fractures. Using age-adjusted rates, we estimate that for every 323 reduction in typical femoral neck or intertrochanteric fractures, there was concurrent increase of 3 subtrochanteric fractures. Even after we included closed femoral shaft fractures, which have been implicated following long-term bisphosphonate therapy,(36) the increase in these hip fractures is still around 10 per 300 typical hip fractures reduced (unpublished data). This estimate was similar to what Black and colleagues reported based on the minimum value of hypothetical relative risk of subtrochanteric or diaphyseal femur fractures in their clinical trial data.

We also confirmed that in the beginning of our study period, subtrochanteric fractures constitute only 2% to 4% of all hip fractures,(37) a proportion of which increased significantly but modestly among women. Thus, even though increasing trends of subtrochanteric hip fractures were significant, the extent of the problem was minor, and the potential for future increases may be limited. However, such incidences should be monitored on the population level and prevented, if possible, during individual care.

In the United States, where burdens of overall hip fractures are falling, annual national estimates and age-adjusted rates of subtrochanteric hip fractures, albeit very low, are on the rise among women aged 65 years and older. Although these increasing trends were temporally correlated with prevalence in bisphosphonate use among postmenopausal women, this study only fulfills a temporality requirement for a possible causal relationship between bisphosphonate use and changes in the patterns and distribution of hip fractures.
