# Supplementary material for: Trends in Incidence of Subtrochanteric Fragility Fractures and Bisphosphonate Use Among the US Elderly, 1996–2007
Source: J Bone Miner Res. 2010 Sep 2;26(3):553–60. doi: 10.1002/jbmr.233 (PMC3179297; doi:10.1002/jbmr.233)
Supplement: Supplementary file 1 [file jbmr0026-0553-SD1.doc]

Medication Use (%)

Medication Use (%)

Medication Use (%)

Medication Use (%)

Glucocorticoids

Beta-blockers

Proton pump inhibitors

eFigure. Prevalence of Other Medication Use in Women and Men.

Year

Year

Year

Year

Statins
